# Multiscale Heterogeneous Fusion for Lightweight Detection of Cotton Growth Stages in Complex Field Imagery

**DOI:** 10.3390/plants15213488

**Published:** 2026-11-08

**Authors:** Chongsen Ma, Xiao Liu, Aimin Luo

**Affiliations:** 1 College of Water Conservancy & Architectural Engineering, Shihezi University, Shihezi 632000, China; 2 Science and Technology on Information Systems Engineering Laboratory, National University of Defense Technology, Changsha 410073, China

**Keywords:** precision agriculture, cotton growth stage monitoring, RT-DETR, RT-HEM-Li fusion

## Abstract

Background: Accurate recognition of cotton growth stages in complex field environments is crucial for optimizing crop management and harvest scheduling. Methods: In this study, a lightweight multiscale heterogeneous fusion detection network, RT-HEM-Li Fusion, was constructed based on the RT-DETR framework to identify five cotton growth stages, namely cotton bud, cotton blossom, early boll, matured cotton boll, and split cotton boll. The network was evaluated on a publicly available cotton growth dataset. To enhance feature representation capability and small-object detection performance, an Inverted Residual Mobile Block was introduced into the ResNet-18 backbone, an adaptive dual-path aggregation pyramid with trident kernels was designed to preserve cross-scale details, and the baseline GIoU loss was replaced with WIoU-Focaler-NWD to enhance small-object localization and class discrimination. Results: RT-HEM-Li Fusion achieved the highest overall mAP@0.5 of 0.782 among the compared models, ahead of TridentNet (0.751) and RT-DETR (0.722) on average, while requiring only 13.905 M parameters and 40.2 GFLOPs, approximately one twentieth of the computational cost of TridentNet and fewer parameters than YOLOv5 (25.048 M), YOLOv8 (25.843 M), and DINO (47.548 M). Conclusions: RT-HEM-Li Fusion provides an effective image-based tool for monitoring cotton growth stages, demonstrating stable detection performance under the complex field conditions represented in the evaluated dataset, with a favorable balance between accuracy and model complexity.

## 1. Introduction

Cotton yield estimation and quality monitoring are crucial for increasing agricultural productivity as cotton is a globally valuable economic crop. Cotton boll development directly affects yield and quality; as such, the developmental stage of cotton must be determined for precision agriculture [1]. In addition, cotton is highly susceptible to pests and diseases. Therefore, dynamic image-based monitoring provides growth information critical for effective pest and disease prevention and control measures in automated farming systems.

Researchers have recently focused on detecting cotton growth. The detection of cotton growth has traditionally and primarily relied on manual inspection [2]. Visual assessment can be affected by observer subjectivity and variability, as documented in studies of plant disease severity assessment [3]. This approach presents several challenges: (1) manual inspection is inefficient as well as labor-intensive and strongly depends on human expertise, leading to high misjudgment rates in maturity assessments, and (2) the abnormal boll settings, caused by global climate change, necessitate high-spatiotemporal-resolution monitoring systems to implement precision agriculture and manage the fields.

Deep learning is showing potential for application in precision agriculture with the rapid advancement of artificial intelligence. Thus, deep learning techniques have been applied to automatically detect the growth stage of cotton, using models such as the faster R-CNN and the YOLO series. Liu et al. proposed MRF-YOLO, a multireceptive field extraction method based on YOLO, to detect and count cotton bolls; the method was validated using cotton farms datasets [1]. Similarly, Zhang et al. improved the YOLOv5 model to accurately and cost-effectively detect unopened cotton bolls by incorporating dense nets, attention mechanisms, and Bi-FPN [4]. Yeom et al. identified open cotton boll candidates based on their spatial growth characteristics and used an automatic thresholding method as well as morphological filters for their binary classification [5]. Song et al. developed an enhanced cascade R-CNN to detect the top buds of cotton in red, green, and blue (RGB) images via integrating the color and depth information from an RGB-D camera to obtain the coordinates of the target in 3D [6]. Lu et al. optimized a YOLOv8-based model through algorithm enhancements and data augmentation techniques to increase detection accuracy under varying lighting and weather conditions [7].

Despite these advancements, existing cotton detection studies mostly target a single reproductive stage or a binary distinction, such as unopened bolls, open bolls, or top buds [8,9,10]. Only a few studies have addressed multiple stages, but their accuracy remains limited and is still short of practical application. Owing to the large differences in object size across stages, the morphological similarity between adjacent stages, and the complexity of field environments, duplicate and missed detections are prone to occur in densely distributed cotton fields.

In view of these challenges, this study designed a novel detection network, RT-HEM-Li Fusion, targeting five cotton growth stages. By introducing an IRMB network, an ADAPT-FPN structure, and a WIoU-Focaler-NWD loss function, the proposed model achieves accurate and lightweight detection of cotton growth stages in complex field environments.

The key contributions of this study are as follows:

(1) Bridging the detection target gap

Most previous studies have focused only on a single stage of cotton growth and development, such as bolling. However, cotton development consists of multiple stages, and different stages of cotton growth, such as flower buds, boll formation, and boll maturity, correspond to key agronomic decisions, such as irrigation scheduling, pest control, fertilizer application, and harvesting scheduling. If only a single stage is monitored, the information obtained will be very limited and critical intervention times may be missed. Therefore, this study aims to identify each growth stage of cotton accurately, which is important for optimizing the agricultural management cycle and improving yield and quality.

(2) Robust Object Detection Model

We developed a heterogeneous, efficient, and multiscale lightweight fusion network (RT-HEM-Li Fusion) to address challenges in image detection including plant occlusion, lighting interference, and leaf reflection in cotton fields. The model integrates an adaptive dual-path aggregation pyramid with trident kernels (ADAPT-FPN), an Inverted Residual Mobile Block (IRMB), and a WIoU-Focaler-NWD loss function to increase the detection accuracy and robustness.

(3) Lightweight and Efficient Design

The RT-HEM-Li Fusion model accurately detects cotton growth stages in complex field environments with relatively low model complexity. The results of ablation studies confirmed its high detection accuracy and stability, and comparative experiments and visualizations demonstrated its detection performance on the evaluated dataset. This study offers methodological references and technical support for the dynamic monitoring of cotton growth stages.

## 2. Discussion

The detection accuracy varied substantially across the five growth stages, and this variation is closely related to the morphology of each stage. Cotton blossoms, with their large, brightly colored petals, and matured cotton bolls, which are large, brown, lignified structures, are the two most visually distinctive stages; these two classes were already well handled by the heavier baseline configuration, and the full model trailed the baseline slightly on them. In contrast, the full model clearly outperformed the baseline on the remaining three stages (Table 3). On these three classes, the full model improved AP@0.5 by 0.050, 0.023, and 0.102, respectively, indicating that the global context captured by the IRMB backbone and the cross-scale details preserved by ADAPT-FPN are most beneficial for small, low-contrast, or easily confused targets. The consistently low AP of early bolls across nearly all compared models (Table 4 and Table 5) further suggests that the transitional morphology of this stage, rather than any specific model design, is the main source of error.

The loss-function ablation (Table 4) shows a similar pattern. Applied alone, WIoU decreased the overall mAP@0.5 from 0.747 to 0.717 while improving only matured cotton boll. A likely explanation is that WIoU down-weights low-quality anchors, and small or low-contrast objects such as buds, blossoms, and early bolls produce a large fraction of low-IoU anchors during training, leaving these classes under-trained. Focaler-IoU restores gradient weight on hard samples, and NWD provides a size-invariant similarity measure tolerant to the large relative position deviations of small boxes; the combined loss therefore achieved the highest and most balanced accuracy, at the cost of a small decrease on matured cotton boll and split cotton boll, which remains acceptable for this morphologically distinctive class. Compared with state-of-the-art detectors (Table 5), RT-HEM-Li Fusion does not dominate every class, TridentNet achieved higher AP on early boll and matured cotton boll, and the baseline RT-DETR on matured cotton bolls, but it achieved the highest overall mAP@0.5 and mAP@0.5:0.95 at a fraction of the computational cost.

## 3. Materials and Methods

### 3.1. Dataset Acquisition

**Figure 1:**
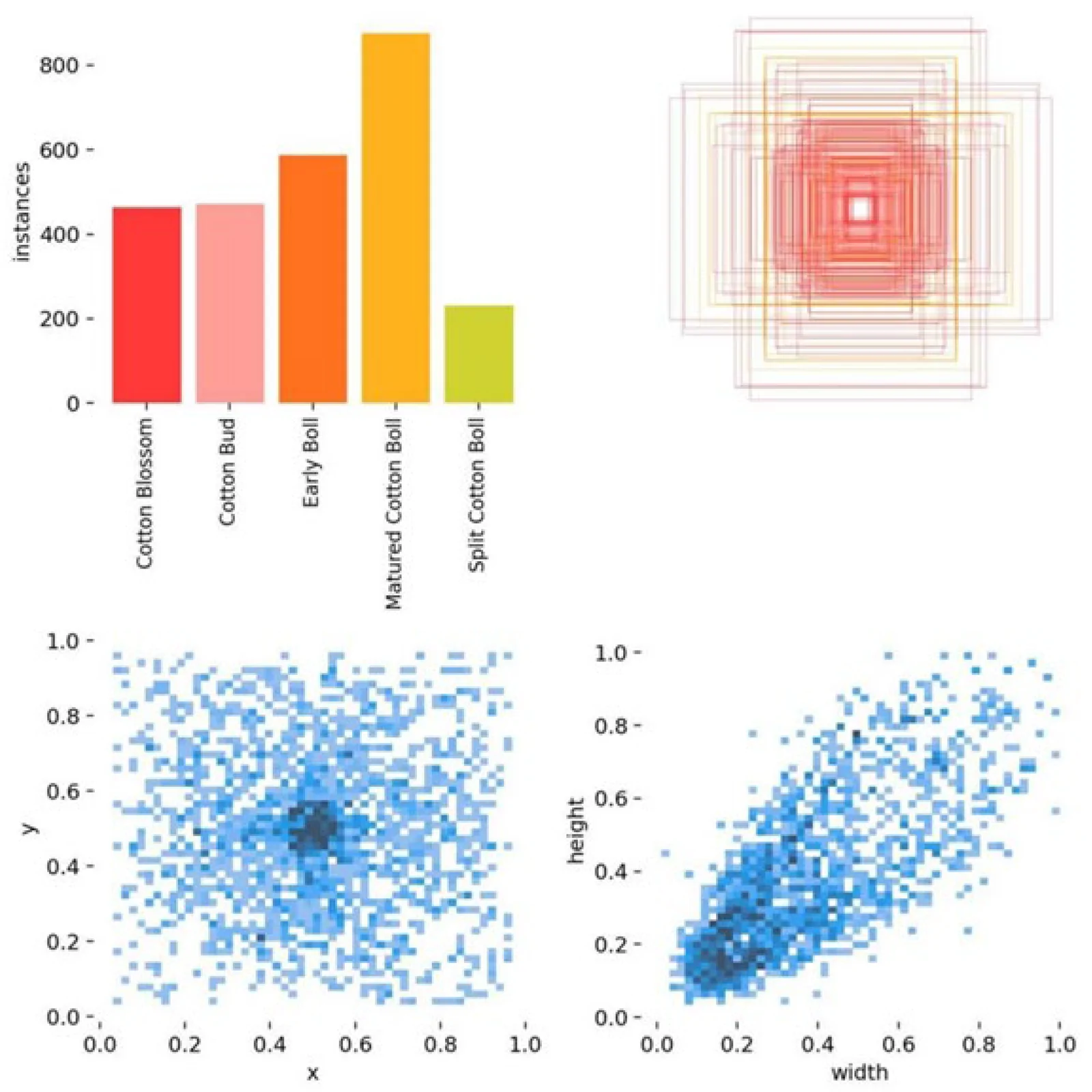
Basic training set information.

The image data were obtained from a publicly available dataset from the RoboFlow Universe website. The dataset included annotated images of various cotton forms during the growth process, including cotton blossoms, cotton buds, early bolls, matured cotton bolls, and split cotton boll, in YOLO format. Only these five target categories were annotated; background elements commonly present in the field images, such as soil, weeds, and plant residues, were left unannotated. The final dataset consisted of 1220, 224, and 61 training, validation, and test images, respectively, in an approximate ratio of 20:4:1, meeting the data analysis requirements. The distribution of the dataset and the image annotations are shown in Figure 1.

**Figure 2:**
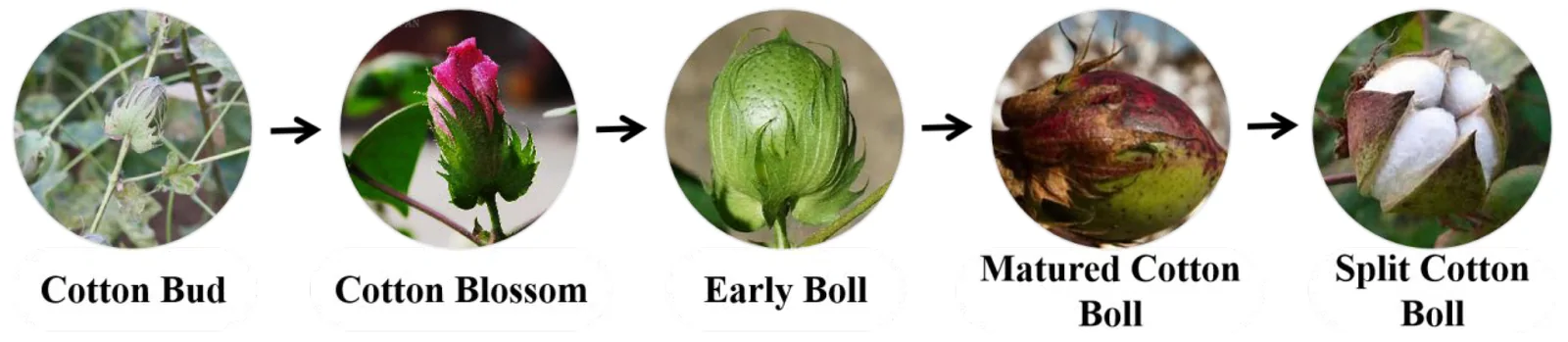
Cotton growth and development process.

The morphological stages of cotton growth and development can be categorized into five primary types (Figure 2): (1) Cotton buds represent the initial stage of reproductive growth and are enclosed within the bracts, appearing as a green conical structure with a diameter of approximately 3–5 mm and containing undifferentiated petals and reproductive structures. (2) The cotton blooms during flowering are off-white or pale yellow, which transitions to pink or red the day before wilting. The center of the flower features a pistil and stamens, and pollination primarily occurs through insects and the wind. (3) The flower withers, and the ovary enlarges, forming a green, spherical cotton boll approximately 2–3 cm in diameter, in the early cotton boll stage, during which the fiber cells rapidly elongate, and seed development begins. (4) The cotton boll ceases to expand, and its outer shell gradually lignifies, in the matured cotton boll stage, with the color transitioning from green to brown. The internal fiber thickening (secondary wall deposition) completes, and the seeds mature and harden. (5) The mature boll splits open along its natural sutures under dry condition, revealing white cotton fibers (boll opening). The cotton segments become dry and fluffy, which facilitate mechanical harvesting.

### 3.2. Methods

#### 3.2.1. RT-DETR

Recent studies have adapted RT-DETR to diverse detection tasks through task-specific architectural improvements. For example, RT-DETR-Tomato enhances tomato detection in complex agricultural environments [11], RS-DETR improves remote-sensing object detection through enhanced attention and multiscale feature fusion [12], and GCD-DETR strengthens multiscale feature extraction and fusion [13].

RT-DETR is a real-time end-to-end object detector developed by the Baidu team designed to balance detection accuracy and computational efficiency [14,15]. RT-DETR introduces multiple optimizations of the DETR architecture, with the primary improvements including the following:

RT-DETR incorporates an efficient hybrid encoder for more effectively processing multiscale features. This encoder comprises attention-based intrascale feature interaction and CNN-based cross-scale feature fusion modules. Specifically, the intrascale feature interaction module applies a single-scale transformer encoder only to the smallest feature map (S5) that contains high-level semantic information to determine intrascale feature interactions. This strategy reduces computational costs and enhances the correlation between objects, thereby increasing precision of localization and recognition. Additionally, convolutional fusion blocks are used in the cross-scale feature fusion module within the feature fusion path to effectively align and integrate cross-scale features and preserve high-quality multiscale feature information.

RT-DETR employs an uncertainty-minimized query selection scheme to address the uncertainty that arises during the object query process in DETR. This approach treats the discrepancy between the spatial distribution of the detected object and the classification results as a joint latent variable of the encoder features, effectively encoding feature uncertainty. This strategy enables the decoder to receive high-quality queries, thereby further increasing the accuracy of object detection.

RT-DETR achieves a balanced trade-off between detection accuracy and inference speed owing to these improvements, which motivated us to adopt RT-DETR as the baseline for cotton growth-stage recognition. However, RT-DETR may still experience the following challenges when detecting different growth stages of cotton in complex field environments, similar to those reported in other agricultural detection tasks [16]:

(1) Imbalanced multiscale object distribution: Cotton boll size widely varies among the growth stages, and the distribution of the object size is imbalanced. This may bias the model toward detecting objects on a certain scale and reduce its accuracy when detecting objects on other scales.
(2) Loss of small-object details: The publicly available dataset contains a large number of small objects, and the fine details of small objects tend to be lost during long-range feature transmission within the RT-DETR network, thereby affecting detection performance.
(3) Environmental complexity and object variability: The cotton growth environment is highly complex with large variations in the characteristics of the object. The detection task involves challenges such as identifying high-density objects under dynamic weather conditions, which can reduce model detection accuracy and robustness.

#### 3.2.2. RT-HEM-Li Fusion

**Figure 3:**
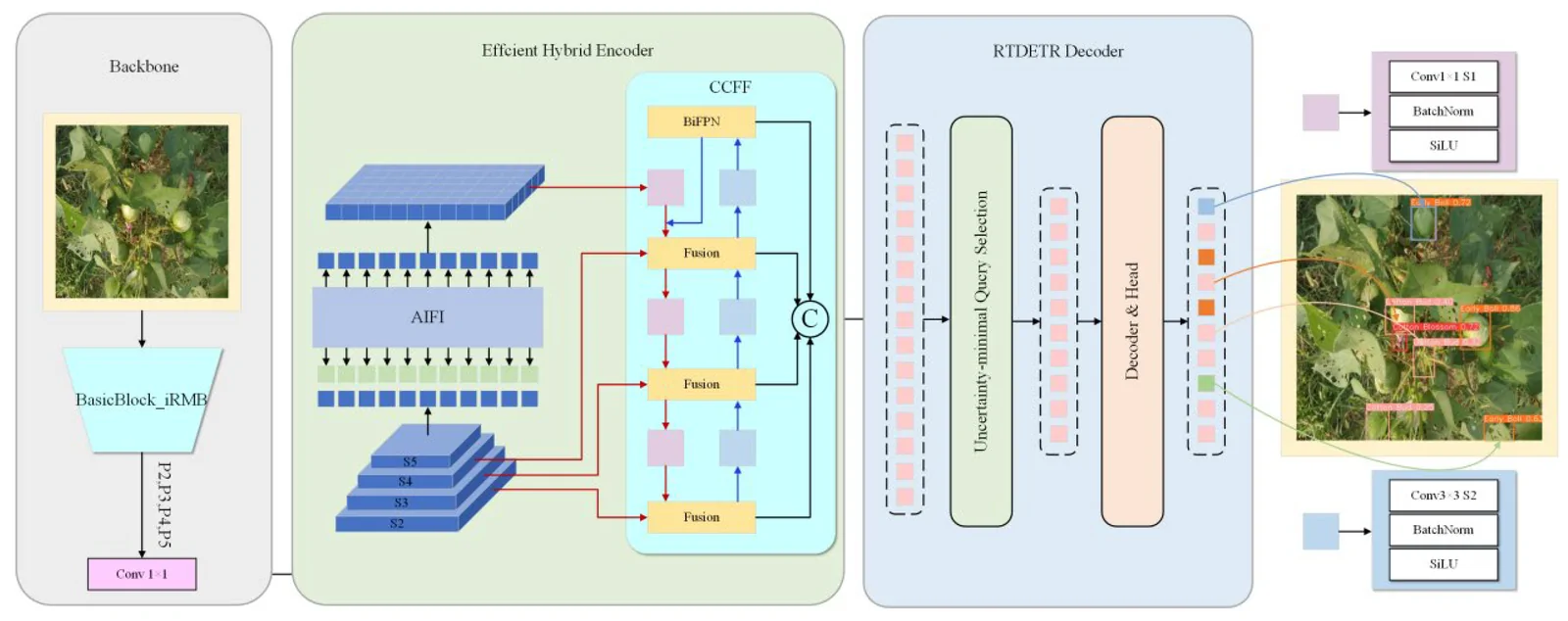
Framework Architecture of RT-HEM-Li Fusion.

The RT-DETR model employs ResNet-18, a traditional CNN architecture, as the backbone for feature extraction [17]. We developed RT-HEM-Li Fusion to address the challenges faced by RT-DETR in detecting different cotton growth stages. The backbone–neck–head structure is adopted as the model architecture for object detection [18,19,20], which consists of three key components: the IRMB backbone, Adaptive dual-path aggregation pyramid with trident kernels (ADAPT-FPN), and head (Figure 3). Specifically, RT-HEM-Li Fusion was built upon the detection framework RT-DETR as a baseline. Then, an IRMB was introduced to optimize the basic block in the ResNet-18 backbone, forming the IRMB backbone. This strengthened the ability of the model to process long-range information while preserving critical global contextual information, thereby preventing local features from overly dominating the model. Next, we integrated a novel multiscale efficient convolution module and a global heterogeneous kernel selection mechanism, inspired by the cross-scale connections in Bi-FPN, leading to the development of the ADAPT-FPN. This module was designed to maximize the retention of small objects and fine-grained details, thereby reducing the loss of small-object features caused by long-range network transmission. Finally, we replaced the generalized intersection over union (GIoU) loss function used in the baseline model with the WIoU-Focaler-NWD loss function. The aim of this substitution was to increase the localization accuracy of small objects as well as object differentiation.

The following sections provide more detailed explanations of these improvements.

#### 3.2.3. IRMB Backbone

The original ResNet-18 faces several challenges when deployed in resource-constrained environments owing to limited storage and computing resources, as well as the network depth and large number of parameters of ResNet-18. In addition, the basic block primarily relies on a local receptive field for feature extraction, hindering the modeling of the global feature dependencies across regions. This limitation weakens the ability of the model to capture long-range dependencies, thereby reducing its representational capabilities in complex environments.

**Figure 4:**
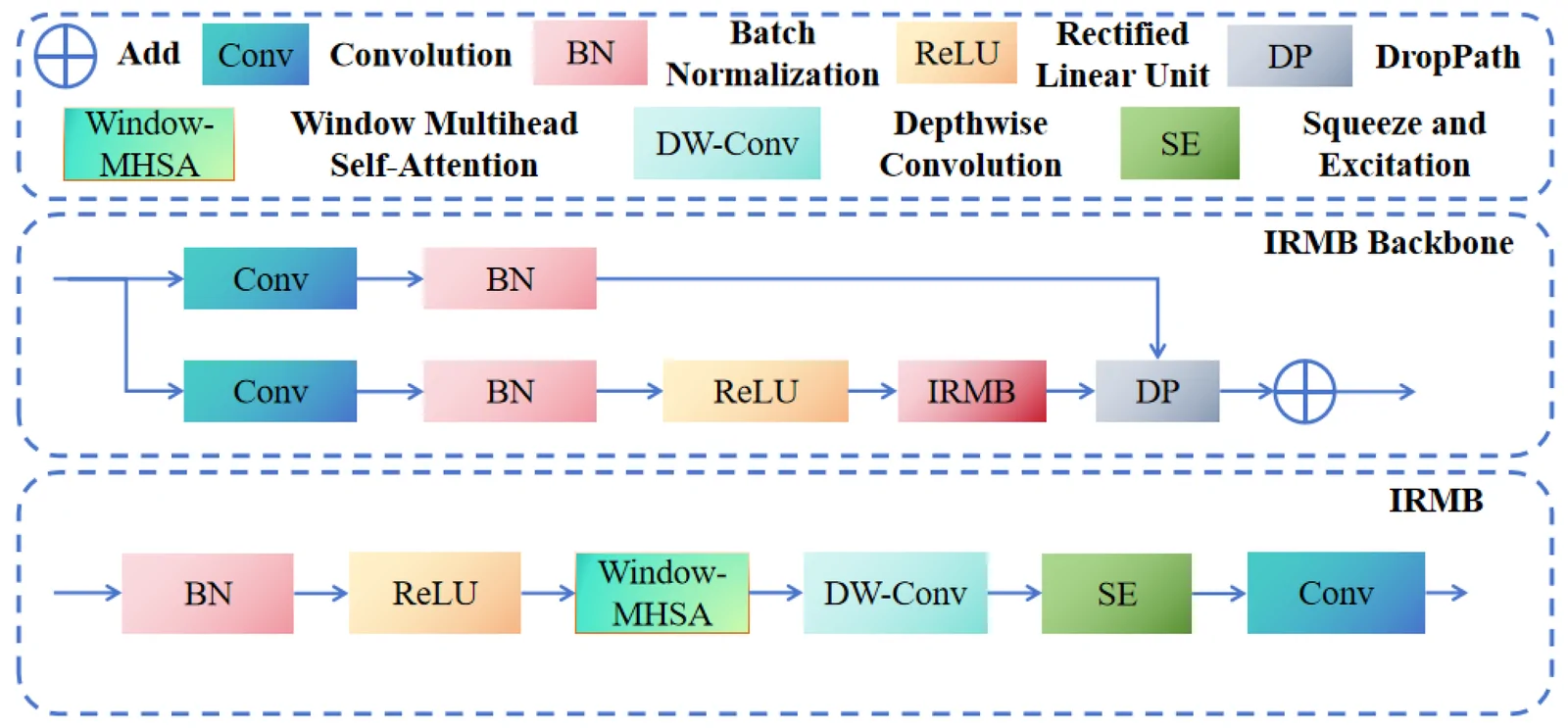
IRMB Backbone Schematic diagram.

We introduced an IRMB to optimize the basic block structure and construct the IRMB backbone to address these issues [21,22]. Our aim was to strengthen the ability of the model to capture global information and reduce computational complexity, providing a model that is more suitable for deep learning applications in resource-constrained environments. The details of the architecture of the IRMB backbone are illustrated in Figure 4.

#### 3.2.4. Adaptive Dual-Path Aggregation Pyramid with Trident Kernels

**Figure 5:**
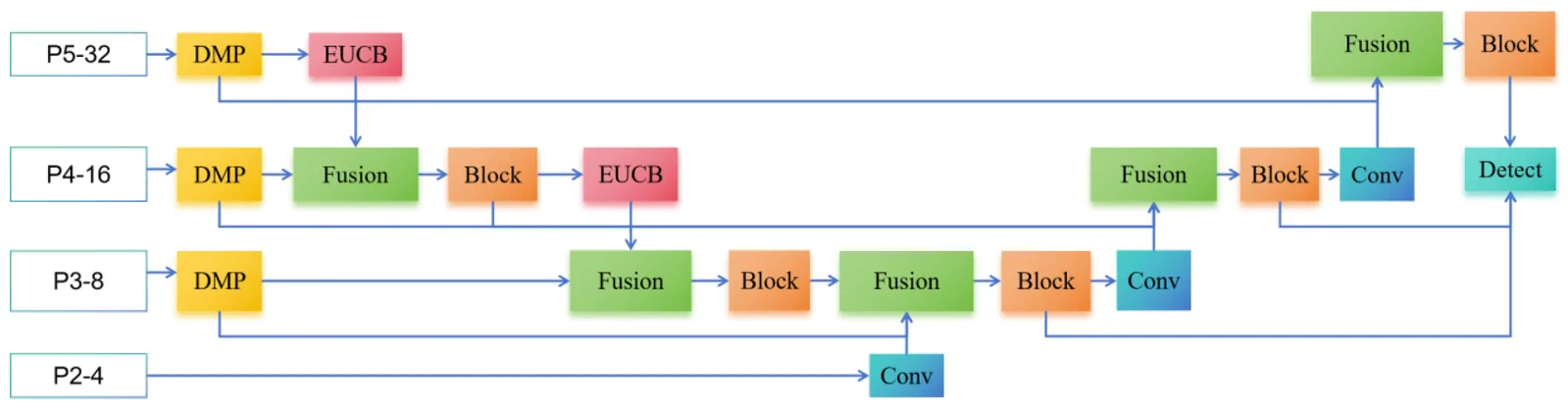
ADAPT-FPN structure.

We focused on the challenge caused by the diversity of the target scale when detecting cotton growth and developmental stages (e.g., the large size difference between cotton buds and matured cotton bolls) using the RT-DETR model. We developed the adaptive dual-path aggregation pyramid with trident kernels (ADAPT-FPN) model that integrates the following key innovations to address this problem:

(1) Multiscale feature-weighted fusion mechanism: We replaced the traditional concatenation operation with addition operations to reduce the computational complexity of the model, inspired by the Bi-FPN [23] cross-scale connection structure. In addition, an adaptive weight allocation mechanism was introduced to enable the dynamic selection of the importance of different scale features.
(2) Dynamic multiscale perceptive field adaptation (DMP): We employed different convolution kernel sizes on various feature map levels based on the trident network framework, enabling the effective detection of cotton growth stages ranging from small to large targets.
(3) Lightweight module design: We incorporated a novel and efficient multiscale convolution module and a global heterogeneous kernel selection mechanism, which enhance the feature representation capability and reduce the number of parameters of the model.
(4) Efficient upsampling strategy: We optimized the upsampling process by leveraging the EUCB module used in the EMCAD [24] method, thereby minimizing the information loss during feature map reconstruction. The detailed ADAPT-FPN structure is illustrated in Figure 5.

#### 3.2.5. WIoU-Focaler-NWD Loss Function

(1) WIoU

The goal in this study was to differentiate the different cotton morphologies during growth and development. Cotton widely differs in shape, texture, and color, posing detection difficulties, which can lead to the model inconsistently detecting various subcategories. Therefore, suitable loss functions are required during the network training process to ensure the effectiveness of the model in detecting various subcategories. However, the traditional GIoU localization loss function of RT-DETR more greatly considers the overlap between the predicted and real frames and not the region between the two, which may lead to biased detection [25].

We replaced the loss function in the original model with WIoUv3 to address this problem. This loss function uses a gradient gain assignment strategy to dynamically optimize the weights of high- and low-quality anchor boxes in the loss function [26,27]. This strategy shifts the center of gravity of the model to average-quality anchor-point boxes, which improves the overall model performance. The specific formulae are provided in Equations (1)–(4) [28,29]:

(1) $${L_{\mathrm{WIoU}}}_{v1}=R_{\mathrm{WIoU}}\times L_{\mathrm{IoU}}$$

(2) $$R_{\mathrm{WIou}}=\exp\left(\frac{{\left(x-x_{\mathrm{gt}}\right)}^{2}-{\left(y-y_{\mathrm{gt}}\right)}^{2}}{\left(W_{g}^{2}+H_{g}^{2}\right)}\right)$$

(3) $$\beta =\frac{L_{\mathrm{IoU}}^{*}}{\bar{L_{\mathrm{IoU}}}}\in \left[0,+\infty \right)$$

(4) $${L_{\mathrm{WIoU}}}_{v3}=r\times {L_{\mathrm{WIoU}}}_{v1},r=\frac{\beta }{\delta \times \alpha^{\beta -\delta }}$$

where IoU is the intersection between the prediction box and the target box that is larger than the union; x,y are the center coordinates of the anchor box; $x_{\mathrm{gt}}$,$y_{\mathrm{gt}}$ are the center coordinates of the target box; Wg and Hg are the width and height of the minimum bounding box, respectively, which are detached from the computed graph (the superscript * denotes this operation); and r is the nonmonotonic aggregation factor.

(2) Focaler

We integrated the Focaler-IoU into the WIoU loss function and constructed Focaler-WIoU to increase the convergence speed and localization accuracy of the bounding box regression loss and thus enhance the generalization ability of the model.

First, the IoU loss is reconstructed using the linear interval mapping method to obtain the focal IoU for optimizing the boundary regression using the following Equations (5) and (6) [30]:

(5) $${\mathrm{IoU}}^{\mathrm{focaler}}=\left\{\begin{array}{cc} 0, & \mathrm{IoU}<d\\ \frac{\mathrm{IoU}-d}{u-d}, & d\leq \mathrm{IoU}<u\\ 1, & \mathrm{IoU}\geq u \end{array}\right.$$

(6) $$L_{\mathrm{Focaler}-\mathrm{IoU}}=1-{\mathrm{IoU}}^{\mathrm{focaler}}$$

where ${\mathrm{IoU}}^{\mathrm{focaler}}$ represents the reconstructed focal IoU, where IoU represents the original intersection over union (IoU) value, and the interval [d, u] falls in the range [0, 1].

The focal IoU was fused to the WIoU loss function (Equation (4)) to construct the new loss function Focaler-WIoU, which is calculated as

(7) $$L_{\mathrm{Focaler}-\mathrm{WIoU}}=r\times R_{\mathrm{WIoU}}\times L_{\mathrm{Focaler}-\mathrm{IoU}}$$

(3) NWD

The traditional loss function usually focuses on the minimum outer rectangle, which may lead to a small overlap area between the predicted and the real bounding box, affecting the target localization accuracy, especially for the detection of small targets, such as cotton growth patterns [31].

We used the normalized wasserstein distance (NWD) as a regression loss calculation index based on the Focaler-WIoU to solve this problem. The specific steps were as follows:

(1) Generate a bounding box based on NWD

The bounding box was modeled using a two-dimensional Gaussian distribution, and the Wasserstein distance was used to calculate the difference between the real and predicted bounding boxes. The position and size of the fake bounding box followed a Gaussian distribution, where the center pixel had the highest weight, which then decreased from the center to the edges. The probability distribution of the predicted box was represented as a parameterized mean and covariance matrix, which was calculated using Equation (8) [32]:

(8) $$W_{2}^{2}\left(N_{a},N_{b}\right)={\left|\left|\left({\left[{\mathrm{cx}}_{a},{\mathrm{cy}}_{a},\frac{\omega_{a}}{2},\frac{h_{a}}{2}\right]}^{2},{\left[{\mathrm{cx}}_{b},{\mathrm{cy}}_{b},\frac{\omega_{b}}{2},\frac{h_{b}}{2}\right]}^{2}\right)\right|\right|}_{2}^{2}$$

where $N_{a}$ and $N_{b}$ denote the Gaussian distributions modeled by the true and predicted frames, respectively. The true and predicted frames are represented by $\left({\mathrm{cx}}_{a},{\mathrm{cy}}_{a},\frac{\omega_{a}}{2},\frac{h_{a}}{2}\right)$ and $({\mathrm{cx}}_{b},{\mathrm{cy}}_{b},\frac{\omega_{b}}{2},\frac{h_{b}}{2}$), respectively.

(2) Normalized Wasserstein Distance (NWD)

Exponential transformation was used to normalize the Wasserstein distance to restrict its value between 0 and 1 as a similarity measure (NWD). The NWD was calculated using Equation (9) [33]:

(9) $$\mathrm{NWD}\left(N_{a},N_{b}\right)=\exp\left(-\frac{\sqrt{W_{2}^{2}\left(N_{a},N_{b}\right)}}{c}\right)$$

The final NWD loss function expression is shown in Equation (10):

(10) $$L_{\mathrm{NWD}}=1-\mathrm{NWD}\left(N_{p},N_{g}\right)$$

where NP is the Gaussian distribution model of the prediction frame P, and Ng is the Gaussian distribution model of the real bounding box G.

(3) Constructing the final WIoU-Focaler-NWD loss function

The weighted combination of the Focaler-WIoU (Equation (7)) and NWD loss (Equation (10)) formed the final WIoU-Focaler-NWD loss function, with the formula shown in Equation (11):

(11) $$L_{WIoU-Focaler-NWD}=\omega \times L_{NWD}+\left(1-\omega \right)\times L_{Focaler-WIoU}$$

## 4. Experiments and Discussions

### 4.1. Experimental Environment Setup

The software and hardware environments used in this study for the training and testing configurations are listed in Table 1.

The initial weights were randomized to ensure that the results reflected the ability of the target detection network to accurately extract the features from the training set to prevent common features from affecting the correlation weights of the proposed target detection network during the training phase. We randomly selected 50% of the images during the training process and mixed them with the corresponding labels to produce a synthetic image. This increased the generality of the model by introducing label noise and visual variations. The image size was normalized to 640 × 640. Table 2 lists the basic training parameters of the model.

### 4.2. Evaluation Metrics

The ability of the proposed detection network to identify different cotton growth stages was evaluated using precision, recall, F1 score, and mean average precision (mAP). Additionally, we computed mAP@0.5:0.95, which is related to mAP@0.5, to more comprehensively assess the detection accuracy. The specific formulations of these metrics are presented in Equations (12)–(15):

(1) Precision

(12) $$Precision=\frac{TP}{TP+FP}$$

where TP indicates the number of true positives, that is, the instances correctly classified as positive; FP is the number of false positives, which indicates the instances that are incorrectly classified as positive. The detection accuracy was calculated in relation to the proportion of TPs among all positively categorized samples.

(2) Recall

(13) $$Recall=\frac{TP}{TP+FN}$$

where FN denotes the number of false negatives, that is, the instances that are incorrectly categorized as negative. Thus, the recall rate represents the proportion of TP samples that are actually positive out of all the samples.

(3) mAP

The mAP is an evaluation metric commonly used in agricultural object detection and is defined as the arithmetic mean of the average precision (AP) across all categories. This metric comprehensively reflects the overall performance of a model. mAP@0.5 and mAP@0.5:0.95 are widely adopted in industry as benchmark indicators.

(1) mAP@0.5 (mAP50) specifically refers to the detection accuracy when the IoU threshold is set to 0.5.
(2) mAP@0.5:0.95 (mAP50:95) represents the mean of mAP values computed over multiple IoU thresholds in the range [0.5, 0.95] with a step size of 0.05. This metric strictly evaluates the model robustness across different scales.

mAP@0.5 and mAP@0.5:0.95 are calculated with Equations (14) and (15), respectively.

(14) $$mAP@0.5=\overset{n}{\underset{i=0}{\sum }}\int_{0}^{1}P_{i}(R_{i})d(R_{i})\left(IOU_{thresh}=0.5\right)$$

(15) $$mAP@0.5:0.95=\overset{n}{\underset{i=0}{\sum }}\overset{0.95}{\underset{j=0.5}{\sum }}\int_{0}^{1}P_{i}(R_{i})d(R_{i})\left(IOU_{thresh}=j\right)$$

### 4.3. Results

#### 4.3.1. Training Results

**Figure 6:**
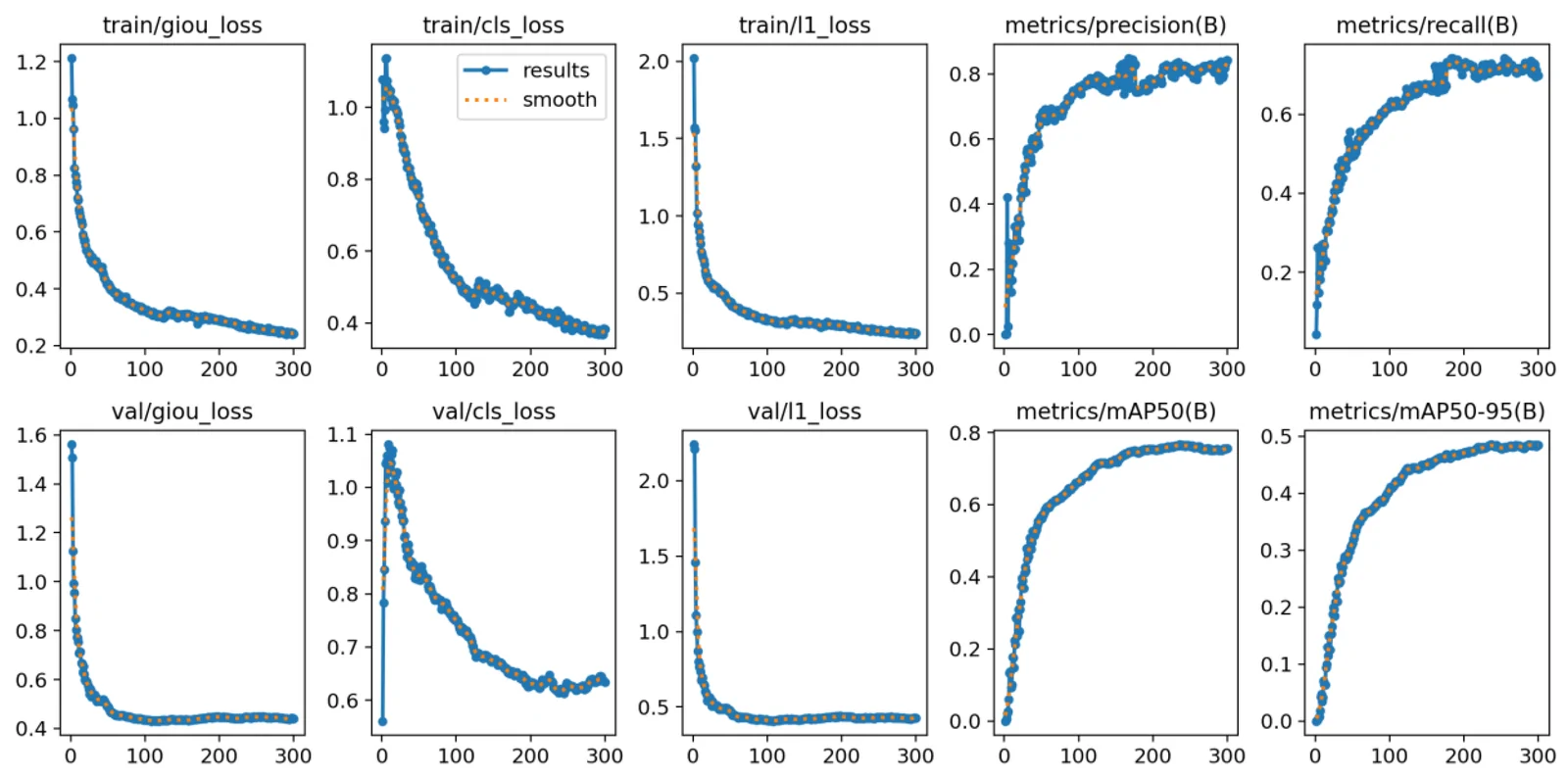
Training loss results.

The RT-HEM-Li Fusion detection network exhibited the following key training dynamics over 300 training cycles (Figure 6):

(1) Loss convergence: The bounding box localization, confidence, and classification losses synchronously decreased with the training cycles, and the loss curves of the training and validation sets converged with the same trend.
(2) Performance saturation: The model precision stabilized after 200 cycles (fluctuation range ±0.3%), and recall peaked at 200 cycles.

#### 4.3.2. Ablation Experiment

Four sets of ablation experiments were conducted using the validation set to further validate the performance of the proposed RT-HEM-Li Fusion detection network as well as to explore the effectiveness of the various improvement methods and the ability of the model to extract information on cotton growth and developmental status from images. The same hyperparameter settings and training cycles were used for each set of experiments; the results are listed in Table 3, where “√” indicates the enabled method or module.

The proposed RT-HEM-Li Fusion detection network performed well across all evaluation metrics (Table 3). The detailed analysis is as follows.

The RT-HEM-Li Fusion detection network achieved the highest mAP @ 0.5 (0.769) on the validation set, with high recognition accuracy, particularly for the cotton blossom and matured cotton boll subcategories. Compared with the baseline RT-DETR, the full model reduces the parameter count to 13.905 M and the computational cost to 40.2 GFLOPs, while improving mAP@0.5 from 0.740 to 0.769; removing either ADAPT-FPN or IRMB degrades performance and increases computational costs, highlighting the balance of accuracy and lightweight design of the model.

Integrating ADAPT-FPN and IRMB produced a high-efficiency network with strong generalization and multiclass recognition capabilities. This model is particularly advantageous for accurately detecting the growth stage of cotton.

#### 4.3.3. Loss Function Analysis

To investigate the effect of the loss function on detection accuracy, we further conducted an ablation analysis of several loss function variants. The results are summarized in Table 4.

As shown in Table 4, RHLF with the original loss function performed stably on most growth stages but was less accurate on cotton bud and split cotton boll. When the original loss was replaced with WIoU alone, the overall mAP@0.5 dropped from 0.747 to 0.717: only matured cotton boll improved, while all other classes declined. This can be attributed to the dynamic focusing mechanism of WIoU, which down-weights low-quality anchors. Small or low-contrast objects such as buds, blossoms, and early bolls produce a large number of low-IoU anchors during training, so these classes receive smaller gradients and are under-trained, whereas matured cotton bolls, as large objects with naturally high IoU, benefit from this mechanism. After Focaler-IoU was introduced, its linear interval mapping restored the gradient weight on hard samples, and the degraded classes largely recovered. NWD was then added on top of this, raising the overall mAP@0.5 to its highest value of 0.782. The combined loss sacrifices a small amount of accuracy on matured cotton boll and split cotton boll in exchange for clear gains on the more difficult classes, and gives the highest and most balanced accuracy across the five stages. This loss configuration was therefore adopted in the following model comparison experiments.

#### 4.3.4. Model Comparison Experiment

We conducted comprehensive comparison experiments to validate the performance of the proposed RT-HEM-Li Fusion target detection network. Specifically, we selected state-of-the-art detection methods for comparison, including DETR, RT-DETR, RT-DETR-L [34], YOLOv5 [35], YOLOv6 [36], YOLOv8, YOLOv9 [37], YOLOv10 [38], YOLOv11 [39], YOLOv12 [40], fast R-CNN [41], Cascade mask R-CNN-R101-FPN [42], TOOD [43], DINO [44], Trident Net [45], and DDOD [46].

DETR and RT-DETR are representative transformer-based detection networks and are widely recognized for their high-quality performance. Fast R-CNN has considerably fewer redundant computations than R-CNN, which increases speed and accuracy. YOLOv5, a mature CNN-based detection model, has been extensively applied in detecting agricultural targets. YOLOv8–YOLOv12, Cascade mask R-CNN-R101-FPN, and Tood are advanced detection networks that exhibit notable detection capabilities. The experimental results are listed in Table 5.

Table 5 provides a comparison of the results obtained with the proposed RT-HEM-Li Fusion target detection network with those obtained with state-of-the-art methods. The RT-HEM-Li Fusion target detection network balanced a lightweight structure with detection accuracy. A specific analysis of the results is as follows:

(1) RT-HEM-Li Fusion achieved the highest overall mAP@0.5 of 0.782, ahead of TridentNet and RT-DETR on average, with AP@0.5 values of 0.920, 0.746, 0.744, 0.798, and 0.704 for cotton blossom, cotton bud, early boll, matured cotton boll, and split cotton boll. It should be noted, however, that the proposed model was not the best on every class: TridentNet achieved higher AP on early boll and matured cotton boll, and the baseline RT-DETR on matured cotton boll. This can be attributed to two factors. First, TridentNet employs three parallel branches with different dilation rates that are specialized for large objects, which favours the large-sized matured bolls, but at a computational cost of 797.2 GFLOPs, about 20 times that of our model. Second, early bolls are a morphologically transitional stage between buds and matured bolls, and their ambiguous class boundaries limit the accuracy of all detectors, including ours.
(2) RT-HEM-Li Fusion has the lowest complexity among the compared transformer-based detectors, with 13.905 M parameters and 40.2 GFLOPs. Inference speed was tested on the device used for training, where the model performed FP32 inference on randomly generated tensors; after 200 warm-up iterations, 1000 consecutive inferences were averaged. RT-HEM-Li Fusion achieved an average inference time of 30.58 ms per image, corresponding to 32.7 FPS, which meets the basic requirement of real-time detection. Limited by the available experimental conditions, inference speed on edge platforms will be further tested in future work.

In summary, the RT-HEM-Li Fusion detection network has considerably fewer parameters, is less computationally complex, and has high detection accuracy, providing strong technical support for high-precision image recognition tasks (e.g., the detection of cotton growth and development stages). Our results demonstrate that the proposed RT-HEM-Li Fusion detection network not only efficiently performs the target detection task of determining the growth and development stage of cotton but also provides competitive performance under a relatively compact model design, which shows potential for use in large-scale, low-resource environments in smart agricultural systems.

#### 4.3.5. Visualization and Analysis of Results

(1) Visualized Comparison of Detection Effectiveness

**Figure 7:**
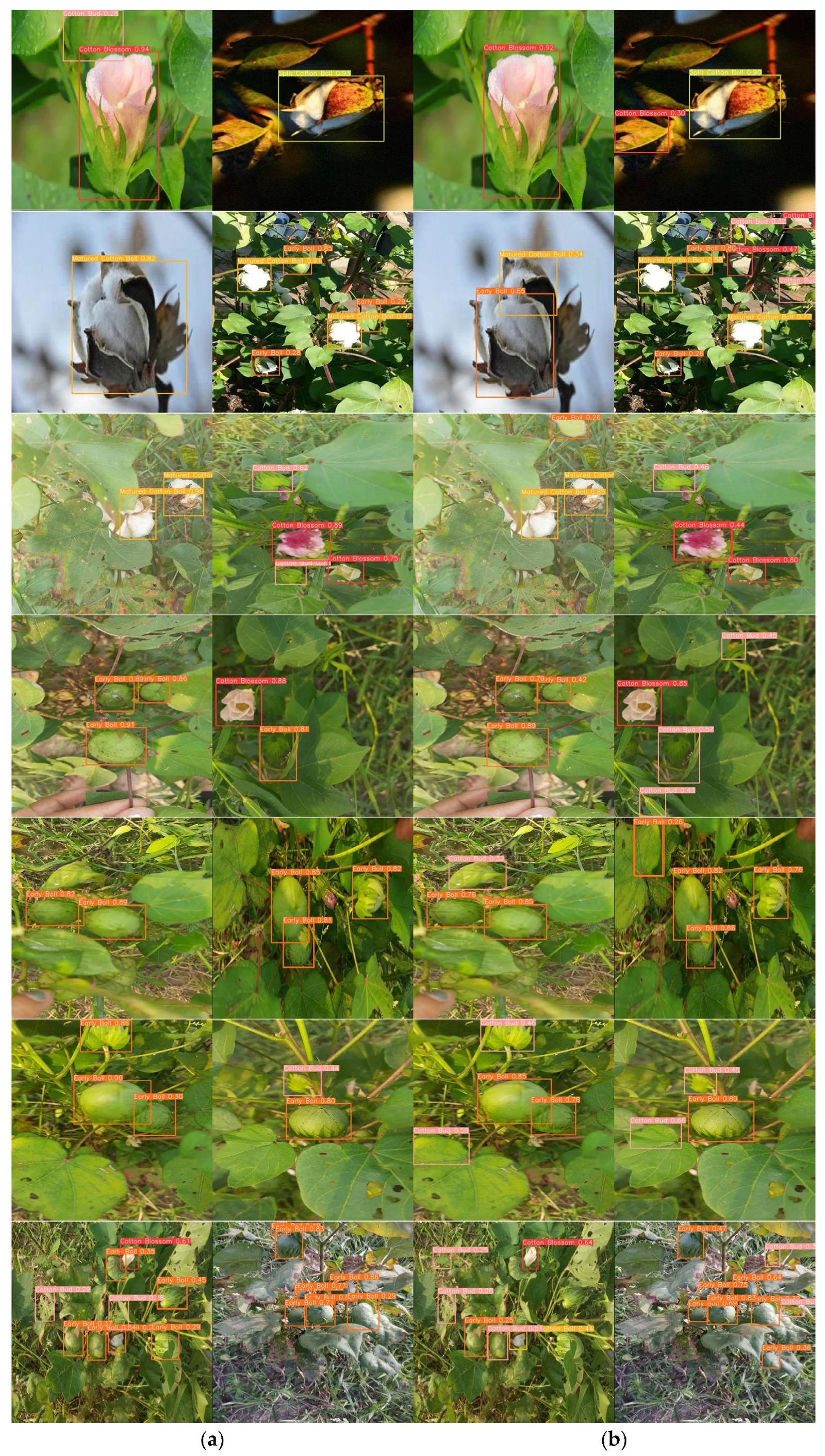
Detection results of (**a**) RT-HEM-Li Fusion and (**b**) RT-DETR.

The detection accuracy, false alarm rate, and missed alarm rate are key evaluation metrics in agricultural target detection tasks that directly indicate the reliability of the model in practical applications. We provide the detection results of the RT-HEM-Li Fusion detection network and the benchmark model RT-DETR to further demonstrate the advantages of the proposed detection network in detecting image information on the growth and developmental status of cotton. RT-DETR misdetected the cotton and early bolls categories (e.g., the second and fifth rows of the first column in Figure 7), whereas the detection results of the RT-HEM-Li Fusion detection network were more accurate, indicating that the proposed network provides advantages in extracting information on the stage of cotton growth and developmental, providing technical support for cotton cultivation and boll picking.

(2) Heatmap

**Figure 8:**
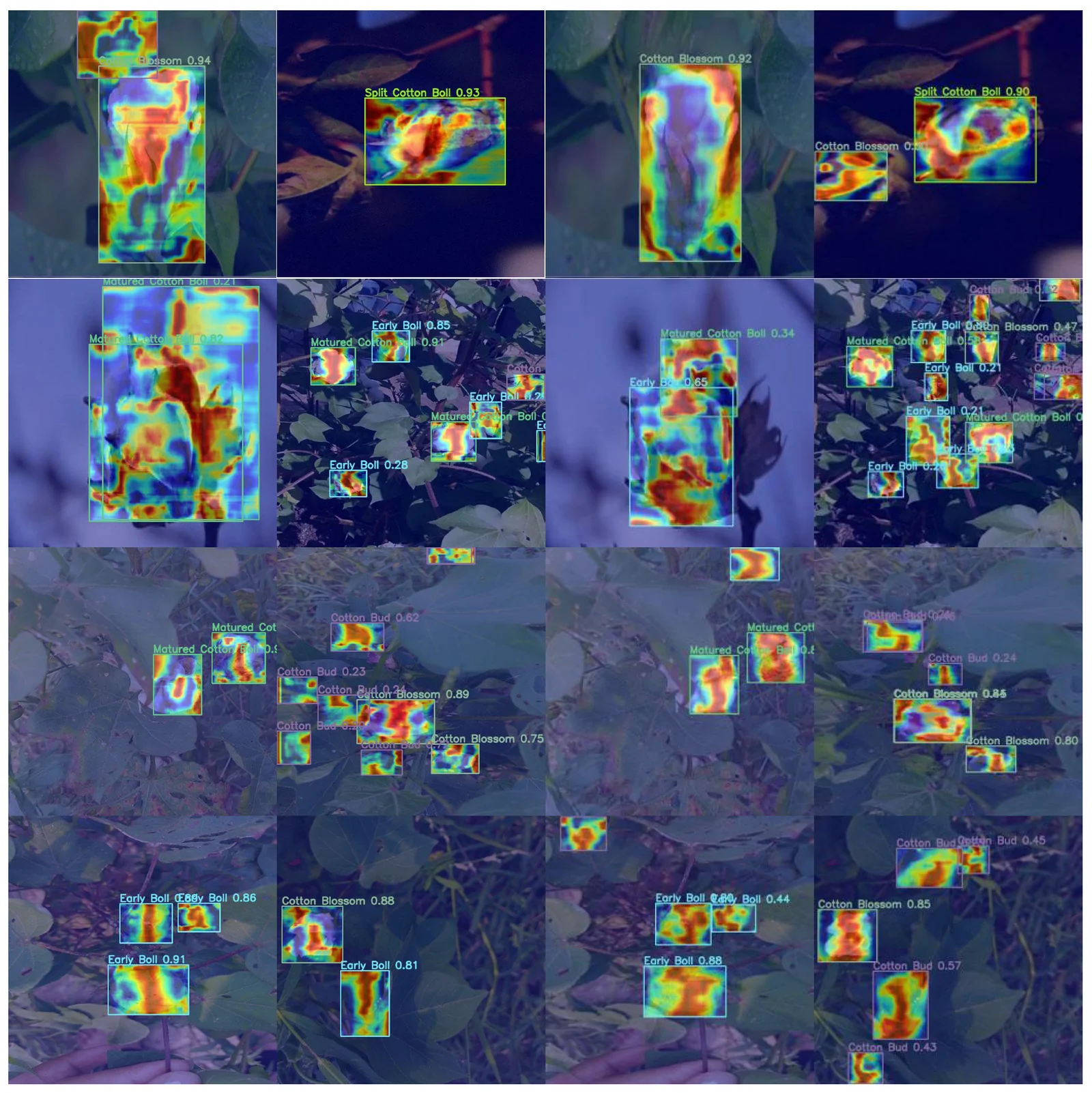

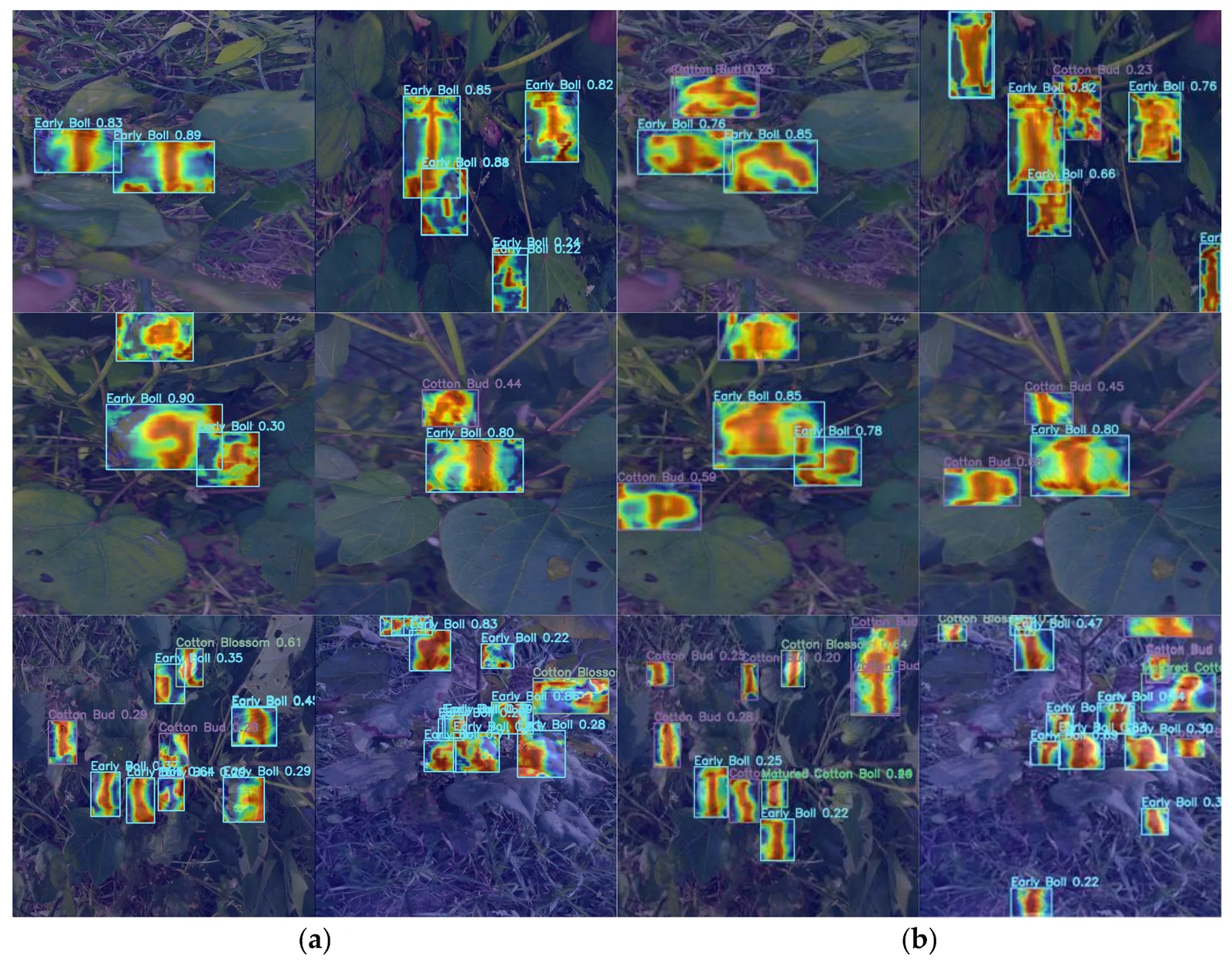
Heatmap results of (**a**) RT-HEM-Li Fusion and (**b**) RT-DETR.

Figure 8 shows the heat maps of the different models in the test set, where red indicates the areas that were important for the model to identify. We only present the heat maps from within the detection bounding box to reduce the interference owing to irrelevant data outside the bounding box. Grad-CAM++ was used for heatmap analysis. Grad-CAM++ has a higher localization accuracy and is more suitable for cases with multiple targets in an image than Grad-CAM [47].

The RT-HEM-Li Fusion detection network more precisely focused on the target objects than RT-DETR, indicating the accuracy of the visualization and detection results (Figure 8). RT-HEM-Li Fusion detection network was more accurate and consistent in identifying high-importance regions, effectively focusing on critical regions and paying less attention to irrelevant regions. These results demonstrate the effectiveness of this detection network and emphasize its superior performance compared with prior networks in complex detection scenarios. Thus, the RT-HEM-Li Fusion detection network is promising for target recognition tasks in complex agricultural environments.

#### 4.3.6. Per-Class Error Analysis

**Figure 9:**
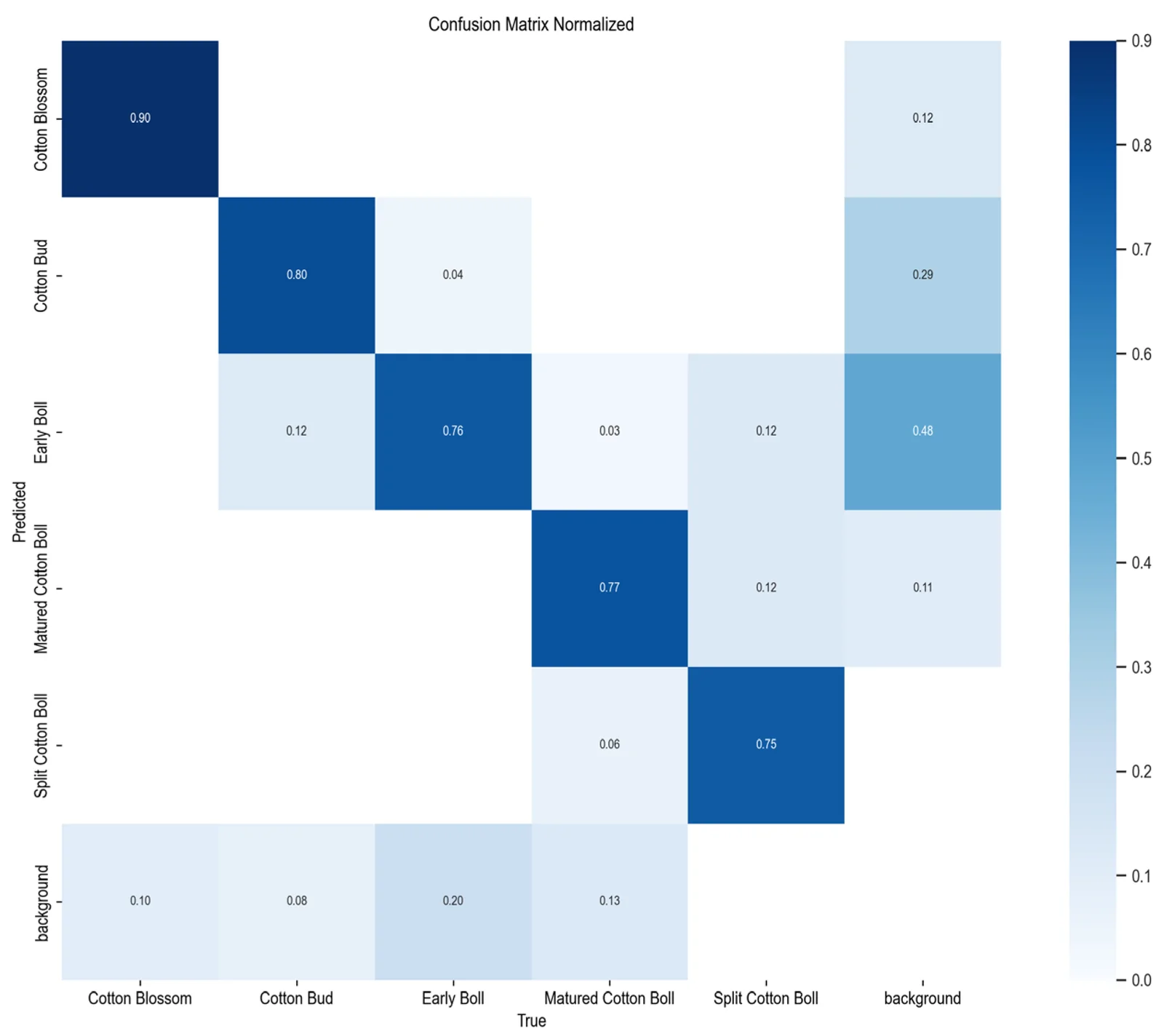
Normalized confusion matrix of RT-HEM-Li Fusion.

To further examine the error patterns of the proposed model across growth stages, we computed the normalized confusion matrix of RT-HEM-Li Fusion on the test set (Figure 9). Per-class recall was 0.90, 0.80, 0.76, 0.77, and 0.75 for cotton blossom, cotton bud, early boll, matured cotton boll, and split cotton boll, respectively. Most classification errors involved the early boll stage: 12% of true cotton buds and 12% of true split cotton bolls were misclassified as early bolls, and early boll also suffered the highest miss rate, with 20% of true instances assigned to background. Confusion between genuinely adjacent stages occurred mainly late in development, where 12% of true split cotton bolls were predicted as matured cotton bolls and 6% of true matured cotton bolls as split cotton bolls; confusion between clearly distinct stages was negligible. This pattern is consistent with the continuous nature of cotton development, in which samples near a stage boundary are inherently ambiguous, and with the similar small, rounded, green appearance shared by buds and young bolls. Among all false detections on background, 48% and 29% were predicted as early bolls and cotton buds, respectively, suggesting that small targets against complex field backgrounds remain the dominant source of error. These results are in line with the design intent of ADAPT-FPN, which preserves small-object details, and help explain the relatively low AP of the early boll and split cotton boll stages reported in Table 4 and Table 5.

#### 4.3.7. Comparison of Effective Receptive Fields (ERFs)

**Figure 10:**
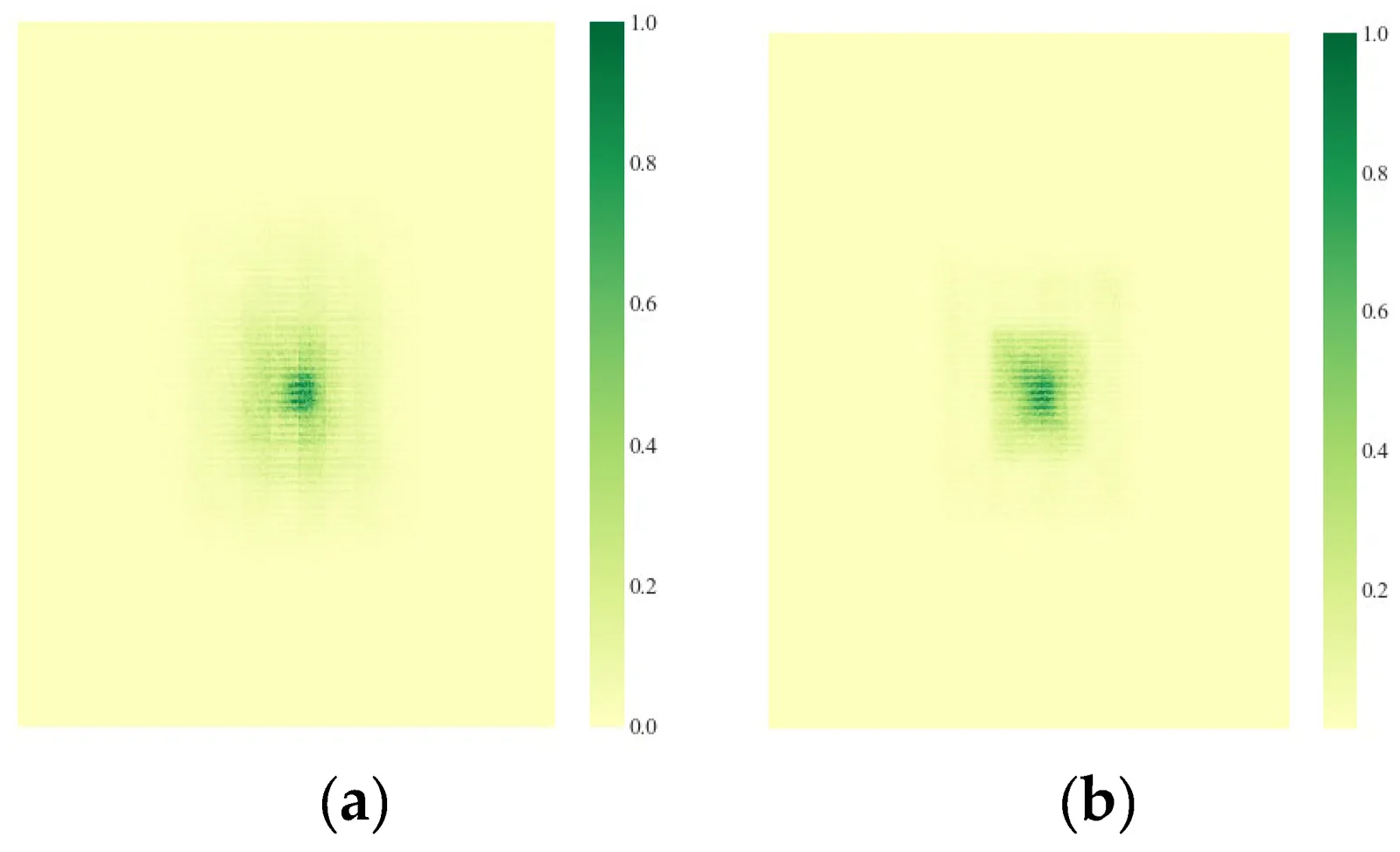
The effective receptive field (ERF) of (**a**) RT-HEM-Li Fusion and (**b**) RT-DETR.

We randomly selected 100 images from the dataset, which we resized to a 1024 × 1024 resolution, to further examine the perceptual abilities of the RT-HEM-Li Fusion detection network. The RT-DETR and RT-HEM-Li Fusion target detection networks were evaluated to determine the ratio of the final feature centroids to the relevant region points in the input images. Figure 10 shows the ERFs of the RT-DETR and RT-HEM-Li Fusion target detection networks. The wider the distribution of dark areas, the larger the ERF. We found that the larger the effective receptive field of the RT-HEM-Li Fusion detection network, the larger its perceptual range compared with that of RT-DETR. This showed that the RT-HEM-Li Fusion detection network receives more comprehensive information and extracts features from images with higher accuracy.

#### 4.3.8. Comparison of Feature Extraction

**Figure 11:**
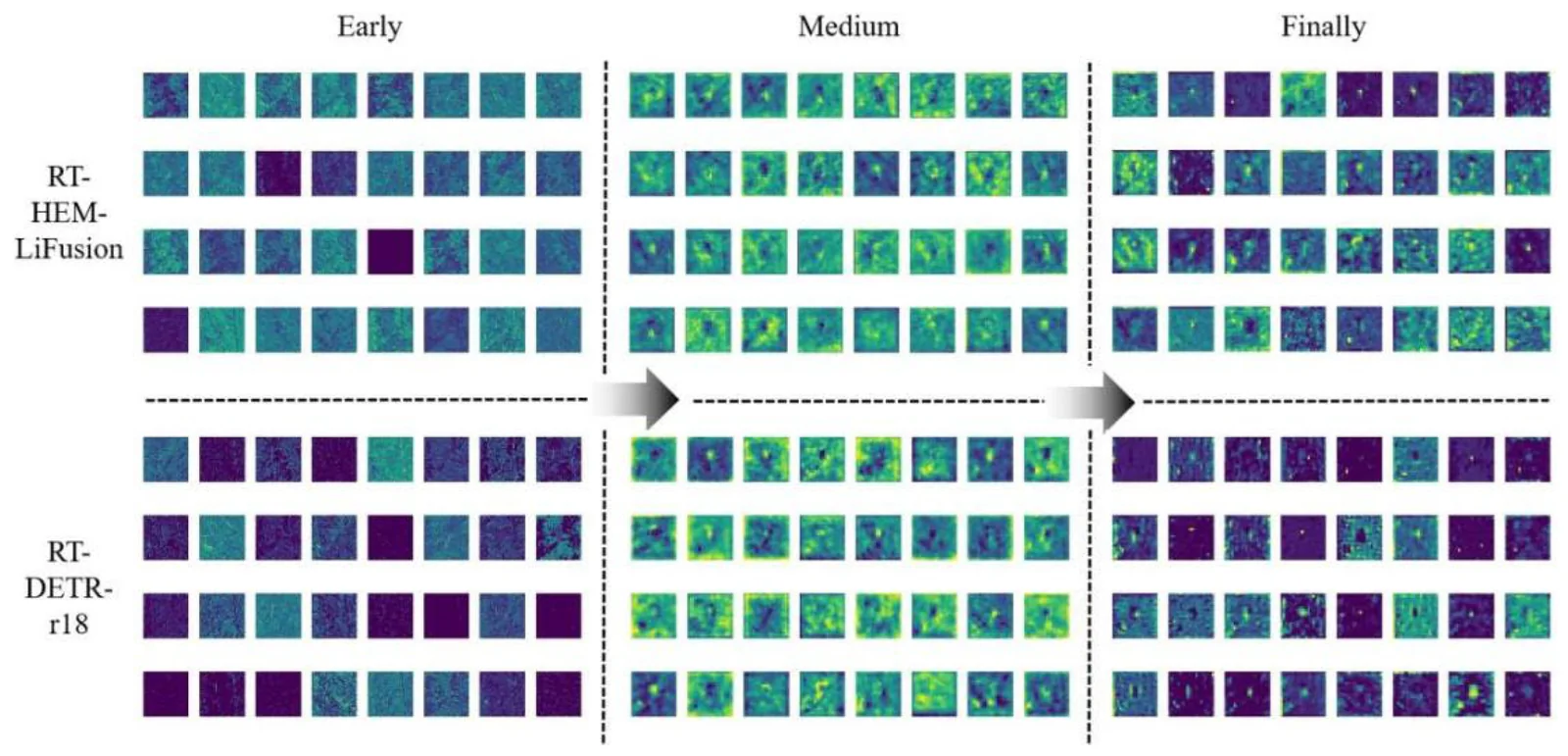
Feature extraction comparison.

We visualize the multistage feature maps of the original RT-DETR and the novel detection network to further demonstrate the feature representation capability of the proposed RT-HEM-Li Fusion detection network in Figure 11. The proposed detection network extracted more morphological cotton growth and development features from the image than RT-DETR.

## 5. Conclusions

We developed the RT-HEM-Li Fusion detection network based on RT-DETR to accurately detect the various growth morphologies of cotton in agricultural fields.

We incorporated the IRMB and ADAPT-FPN strategies into our proposed model to address the challenges involved in the detection of small targets and complex backgrounds in crop images. These modifications allow the proposed model to preserve small objects, mitigate the feature loss caused by long-distance network transmission, and increase localization accuracy.

The RT-HEM-Li Fusion network achieved the highest overall detection accuracy among the compared models while requiring only 13.905 M parameters and 40.2 GFLOPs, demonstrating a favorable trade-off between accuracy and efficiency across the five cotton growth stages. Moreover, the model achieved stable performance on the complex field imagery of the evaluated dataset, including scenes with occluded and overlapped targets, where it outperformed the baseline RT-DETR. The proposed method would add value to smart farmland management.

RT-HEM-Li Fusion provides an efficient and automated tool for cotton growth stage detection, and its lightweight multiscale design may serve as a methodological reference for other small-target crop detection tasks.

While the proposed model achieved promising results on the evaluated public dataset, several limitations should be noted: the test set is relatively small, external validation on an independent dataset was not performed, robustness to out-of-class field objects such as weeds was not systematically evaluated, and inference speed on edge platforms remains untested. These aspects are identified as directions for future work.

In the future, we will focus on validating the model on larger and more diverse datasets, evaluating its robustness to out-of-class field objects such as weeds, benchmarking its inference speed on edge computing platforms, and exploring self-supervised learning to facilitate its adaptation to diverse growing regions and climatic conditions.

## Figures and Tables

**Table 1:** Software and hardware used in this study.

Configuration	Parameter
CPU	13th Gen Intel(R) Core(TM) i7-13700
GPU	GeForce RTX3070TI@8G
Memory Capacity (GB)	32
Training Environment	CUDA12.1
Operating System	Windows 11
Development Environment	Python 3.8.19 Pytorch2.1.0 scipy 1.10.1 numpy 1.24.4

**Table 2:** Basic model training parameters.

Model Parameter	Value
Epochs	300
Batch size	4
Workers	0
Seed	42
Optimizer	AdamW
IoU	0.7
Lr0	0.0001

**Table 3:** Results of ablation experiments.

	ADAPT-FPN	IRMB	Parameters (M)	GFlops	P	R	mAP 50	AP@0.5		mAP @0.5:0.95
1	√	√	13.905	40.2	0.804	0.725	0.769	Cotton blossom	0.888	0.486
								Cotton bud	0.678	
								Early boll	0.578	
								Matured cotton boll	0.852	
								Split cotton boll	0.846	
2	√	×	18.496	50.3	0.813	0.671	0.724	Cotton blossom	0.873	0.462
								Cotton bud	0.627	
								Early boll	0.554	
								Matured cotton boll	0.833	
								Split cotton boll	0.735	
3	×	√	16.629	50.5	0.682	0.899	0.72	Cotton blossom	0.884	0.47
								Cotton bud	0.602	
								Early boll	0.571	
								Matured cotton boll	0.843	
								Split cotton boll	0.7	
4	×	×	19.878	57.0	0.846	0.701	0.74	Cotton blossom	0.901	0.492
								Cotton bud	0.628	
								Early boll	0.555	
								Matured cotton boll	0.872	
								Split cotton boll	0.744	

**Table 4:** Results of Loss function improvement experiment.

Method	mAp@0.5	Ap@0.5				
		Cotton Blossom	Cotton Bud	Early Boll	Matured Cotton Boll	Split Cotton Boll
RHLF (unchanged loss function)	0.747	0.948	0.592	0.72	0.81	0.663
RHLF + WIoU	0.717	0.89	0.575	0.657	0.837	0.625
RHLF + WIoU-Focaler	0.773	0.911	0.675	0.729	0.827	0.721
RHLF + WIoU-Focaler-NWD	0.782	0.92	0.746	0.744	0.798	0.704
RHLF + WIoU-Inner-NWD	0.692	0.916	0.585	0.692	0.833	0.431

**Table 5:** Results of comparison of prior with proposed models.

Methods	mAp@0.5	Ap@0.5					mAP @0.5:0.95	No. Param	GFlops
		Cotton Blossom	Cotton Bud	Early Boll	Matured Cotton Boll	Split Cotton Boll			
RHLF	0.782	0.92	0.746	0.744	0.798	0.704	0.491	13.905 M	40.2
YOLOv5	0.573	0.765	0.364	0.468	0.757	0.513	0.34	25.048 M	64
YOLOv6	0.467	0.619	0.362	0.333	0.486	0.536	0.259	51.98 M	161.2
YOLOv8	0.693	0.881	0.473	0.678	0.741	0.69	0.442	25.843 M	78.7
YOLOv9	0.6	0.787	0.43	0.359	0.771	0.652	0.336	20.017 M	76.5
YOLOv10	0.647	0.782	0.394	0.632	0.735	0.693	0.394	15.316 M	58.9
YOLOv11	0.594	0.713	0.464	0.511	0.755	0.529	0.352	20.033 M	67.7
YOLOv12	0.65	0.815	0.407	0.479	0.781	0.766	0.394	20.109 M	67.1
FR	0.585	0.913	0.163	0.446	0.823	0.581	0.341	28.684 M	101.715
CMR	0.321	0.608	0.124	0.130	0.519	0.224	0.177	96.028 M	1753.926
TOOD	0.255	0.245	0.166	0.248	0.326	0.293	0.137	32.028 M	123.423
DINO	0.600	0.737	0.506	0.410	0.760	0.586	0.364	47.548 M	178.525
Trident-net	0.751	0.910	0.529	0.785	0.828	0.704	0.457	33.076 M	797.210
RT-DETR-L	0.719	0.841	0.598	0.665	0.775	0.718	0.455	31.994 M	103.5
RT-DETR	0.722	0.916	0.559	0.686	0.817	0.631	0.477	19.878 M	57
DDOD	0.298	0.559	0.167	0.059	0.577	0.130	0.139	32.378 M	113.659

RHLF denotes the proposed RT-HEM-Li Fusion target detection network, FR denotes fast R-CNN, and CMR denotes Cascade-Mask-R-CNN R101-FPN.

## Data Availability

Data will be made available on request.

## Abbreviations

The following abbreviations are used in this manuscript:

IRMB	Inverted Residual Mobile Block
ADAPT-FPN	Adaptive Dual-path Aggregation Pyramid with Trident Kernels
GIoU	Generalized intersection over union
RT-DETR	Real-time detection transformer
RT-HEM-Li Fusion	Real-time, heterogeneous, efficient, and multiscale lightweight fusion network
YOLO	You only look once
CNN	Convolutional neural network
R-CNN	Region-CNN
Bi-FPN	Bi-directional Feature Pyramid Network
